# Physical Activity, Body Mass Index (BMI) and Abdominal Obesity of Pre-Adolescent Children in the Region of Thrace, NE Greece, in Relation to Socio-Demographic Characteristics

**DOI:** 10.3390/children9030340

**Published:** 2022-03-02

**Authors:** Niki Dampoudani, Athanasia Giakouvaki, Despoina Diamantoudi, Georgia Skoufi, Christos A. Kontogiorgis, Theodoros C. Constantinidis, Evangelia Nena

**Affiliations:** 1 Laboratory of Hygiene and Environmental Protection, Medical School, Democritus University of Thrace, 68100 Alexandroupolis, Greece; damboudani@gmail.com (N.D.); georgiaskoufi@yahoo.gr (G.S.); ckontogi@med.duth.gr (C.A.K.); tconstan@med.duth.gr (T.C.C.); 2 Primary Education Directorate, Ministry of Education and Religious Affairs, 15180 Marousi, Greece; athanasiagiak@yahoo.gr; 3 Medical School, Democritus University of Thrace, 68100 Alexandroupolis, Greece; ddiamant@admin.duth.gr; 4 Laboratory of Social Medicine, Medical School, Democritus University of Thrace, 68100 Alexandroupolis, Greece

**Keywords:** pre-adolescents, schoolchildren, physical activity, obesity, BMI, socio-economic characteristics

## Abstract

This study aimed to explore the prevalence of overweight, obesity, and abdominal obesity, and define predictive factors of their occurrence among pre-adolescents in the region of Thrace, NE Greece. A secondary aim was to record physical activity at different times (schooldays, weekends, holidays) and explore associations with characteristics such as gender, overweight and obesity, and socio-demographic conditions. A cross-sectional study was conducted involving children aged 11–12 years. Participating children were measured for height, weight, and waist circumference, and their parents answered a questionnaire. In total, 1929 children were included. Prevalence of overweight was 31.5%, prevalence of obesity 17%, and only 1% of children were underweight. Abdominal obesity was detected in 20.3% of the sample. Overweight and obesity were more frequent in males, who also had higher mean values of Body Mass Index-for-age z-scores (BMIaz) and Waist-to-Height ratio (WtHR). Obesity rates were higher among those not participating in physical activities; however, no significant difference was observed between normal-weight, overweight and obese children in the time spent for organized athletic activities or free play. Logistic regression analysis showed that the probability for overweight/obesity was higher in boys (OR = 1.39, 95% CI = 1.16–1.66) and lower in children whose fathers had a higher educational level (OR = 0.75, 95% CI = 0.60–0.93). The probability for abdominal obesity was also higher in boys (OR = 1.37, 95% CI = 1.10–1.72) and lower in children participating at least one hour/day in an organized physical activity (compared to those with no physical activity (OR = 0.66, 95% CI = 0.51–0.85), and whose father was exercising at least 1–2 times/week (OR = 0.76, 95% CI = 0.591–0.98). In conclusion, the prevalence of overweight and obesity among pre-adolescents in NE Greece was estimated at 48.5%. This is associated with the male gender and not participating in physical activities. The educational level and exercise habits of the father (but not of the mother) affect the probability of obesity and abdominal obesity, respectively.

## 1. Introduction

“More active people for a healthier world” is the global action plan for physical activity 2018–2030 of the World Health Organization (WHO) [1]. Indeed, physical activity is associated with improving the physical and mental health of children and is essential for healthy development. It is also the basis for creative employment and motivates the establishment of exercise as a way of life [2,3]. Regular physical activity can contribute to lifelong health and wellness [4]. One aspect that raises concerns about the fitness levels of children is the progressive increase in overweight and obesity [5]. Obesity occurs due to a complex interaction between genetic and external factors, including diet, exercise, and sleep [6].

The prevalence of childhood obesity is steadily increasing globally [7,8]. This is an issue of public health concern, as obese children are at a higher risk of becoming obese adults [9] and eventually developing cardiovascular and metabolic disorders [10]. Regarding cardiovascular risk, it has been previously reported that it can be more accurately predicted by abdominal obesity compared to BMI as a sole index [11].

Unhealthy behaviors, such as low levels of physical activity, increased screen time, poor diet, and short sleep duration have been associated with cardiovascular disease, obesity, and other adverse effects on children’s health [12]

Starting from school age, moderate to vigorous physical activity (MVPA) decreases, and sedentary behavior increases in both genders, resulting in large percentages of older children not meeting the recommended MVPA [3,13]. It has been previously shown that family, school, and community environments correlate with children’s physical activity and body weight [1].

A first step to reaping the potential benefits of the above correlations could be to explore possible correlations of socio-economic status with parameters such as physical activity, daily activities, diet, and sleep, especially those that are potentially modifiable [2,14]. In this context, parental modeling is an important social factor that influences the development of active and sedentary habits in young people [15]. It has been shown, for example, that the larger the living space and the higher the parental education, the lower the children’s BMI [16]. Additionally, the parents working long hours has been associated with certain behavioral patterns in schoolchildren aged 9–11 years [17].

Special focus should be placed on the transition from childhood to adolescence period, as it is associated with multiple changes in behaviors. Additionally, a higher level of decision-making autonomy, along with health-related cognitive development, is observed. In most cases, compliance with the recommendations may be influenced by social, cultural, or economic factors [18].

The aim of this study was to focus on this sensitive period of child growth by exploring the prevalence of overweight, obesity, and abdominal obesity and defining those factors that could predict the occurrence of those characteristics in our study sample. A secondary aim was to record physical activity at different times (schooldays, weekends, holidays) and explore associations with characteristics, such as gender, overweight and obesity, and socio-demographic conditions.

## 2. Materials and Methods

### 2.1. Study Design

This is a cross-sectional study that took place between April 1, 2016, and April 18, 2018. Initially, official approval was obtained by the Hellenic Ministry of Education and Religious Affairs-(Protocol Nr.: F15/284/35997/DΙ/1-3-2016) and the Institutional Review Board (DUTH/TIATR/9256/728/11-3-2016).

Out of the 126 schools in the region, 77 were randomly selected, using a random number generator applied in the official list of the Ministry of Education. This list included all primary schools of each of the three prefectures in the region.

Inclusion criteria involved the following: First, the consent from the school principal to facilitate conducting the study. Regarding participants, inclusion criteria involved the possession of an Individual Student Health Card, the signed consent of the parent or guardian, and the child’s consent throughout the process.

### 2.2. Study Procedure

As a first step, an informative campaign concerning the purpose, the procedure, and the expected results of the study, targeting Heads of the three Directorates of Primary Education, as well as the School Counselors in the region (prefectures of Evros, Rodopi, and Xanthi) was organized. Following that, visits were made to the schools, after consultation with the principals and teachers at each school unit, in line with the school program to inform them about the aim, procedure, and expected gains of the study. At the end of the visit, closed envelopes addressed to parents/guardians of the children were distributed. Each envelope included the following: (1) Informative letter for parents/guardians, describing the purpose of the study, the procedure, and the expected results-providing also information for correspondence (2) Statement of consent to be signed by the parent or guardian, (3) A questionnaire to be answered, (4) A clean envelope for the answered questionnaire to be included, to ensure sensitive data protection.

According to the study protocol, the students had to return both the questionnaire (in a closed envelope) and the statements of consent to be eligible for body measurements. These were collected during physical education class on the days when the researchers visited the school units.

### 2.3. Participants

According to the random selection procedure, 77 public primary schools in the region of Thrace were initially selected. Out of them, 53 school principals responded to the invitation and contacted the research team to schedule a school visit, according to the above-mentioned protocol. The total number of 5th and 6th grade students in the participating schools was 3317.

### 2.4. Body Measurements—Anthropometric Indicators

To assess the children’s physique, height, weight, and waist circumference measurements were performed during school visits. The measurements were made during the Physical Education lesson and always in the presence of the teacher of each class. The body measurements were performed according to the “Guide to the Evaluation of Physical Fitness & Fitness for students of Kindergarten, Elementary, High School & Lyceum” of the program EYZHN (National Health Action for Youth Life), 2014–2015. The equipment used included a tape measure, paper tape, ruler, and electronic precision balance (Bosch-PPW2250). Measurements were conducted only on children with a signed consent form. To ensure the anonymity of the enclosed questionnaire and at the same time to match measurements with the questionnaire results, encoded measurements were written on the back side of the envelope.

Body Mass Index was calculated based on measurement of weight (measured in Kilograms) and height (measured in meters), using the formula:

$$\mathrm{BMI}=\frac{\mathrm{Weight}}{{\left(\mathrm{Height}\right)}^{2}}\;(\mathrm{kg}/m^{2})$$

Following that, the WHO-AnthroPlus v.1.0.4 (available at https://www.who.int/toolkits/growth-reference-data-for-5to19-years/application-tools (accessed on 23 February 2022) was used to calculate BMI-for-age z-scores (BMIaz) and BMI-for-age percentiles (BMIap). Based on BMIaz values and proposed cut-offs, children were divided into 4 groups [19].

(1) Underweight: BMIaz ≤ −2
(2) Normal weight: −2 < BMIaz < 1
(3) Overweight: 1 ≤ BMIaz < 2
(4) Obese: BMIaz ≥ 2

For the evaluation of abdominal obesity, the waist circumference to height, both measured in centimeters, (Waist-to-Height Ratio, WtHR), was used. A WtHR value ≥0.5 has been previously proposed as a reliable index of abdominal adiposity, i.e., fat distribution in the abdominal area, which identifies children at a greater risk for developing cardiovascular or metabolic disorders [20,21]

### 2.5. Questionnaire

The distributed questionnaire was answered by consenting parents or guardians. It was divided into two parts:

Part A: Socio-demographic characteristics: This included several open-ended or multiple-choice questions concerning socio-demographic information, namely place of residence (urban, semi-urban, rural), the structure of the family (e.g., single-parent family, living with grandparents, etc.), educational level, and physical activity habits of both parents.

Part B: Physical Activity characteristics. This part included multiple-choice questions and Likert scales (ranged from 1–10) regarding a child’s preference for sports activities. Additionally, sedentary behaviors and physical activity duration on school days, weekends, and school holidays were explored.

### 2.6. Statistical Analyses

Categorical variables are presented as absolute (n) and relative frequencies (%), while quantitative variables as mean ±standard deviation. Kolmogorov-Smirnov test and Q-Q Plots were used to control the normal distribution of quantitative variables. The Pearson’s Chi-square Test of Independence (χ^2^ test) was used to investigate the difference between two categorical variables, while the parametric t-test for independent samples was used between a quantitative variable, following the normal distribution and a bisector variable. If the quantitative variable did not follow the normal distribution, the non-parametric Mann-Whitney U Test was applied. Multiple logistic regression analysis models were applied to explore predictive variables for obesity and abdominal obesity. In this case, results are displayed as odds ratios (OR), together with 95% confidence intervals (95% CI) and *p*- values.

The bilateral level of statistical significance for the present survey was set equal to 0.05. Data analysis was performed using SPSS 22.0 software.

## 3. Results

### 3.1. Socio-Demographic Characteristics

According to the random selection procedure, 77 public primary schools in the region of Thrace were initially selected. Of them, 53 school principals responded to the invitation and were enrolled in our study. Of the 3317 eligible students, 1929 questionnaires were gathered.

The sample comprised pre-adolescents (age 11–12 years) of the last two classes of the primary school. Those were distributed almost equally, i.e., 50.2% from the 5th grade and 49.8% from the 6th grade. The majority of students lived in urban areas (75.8%) and only 12.5% in rural settings. Regarding gender distribution, the majority (53%, n = 1022) were girls and 47% (n = 907) were boys. The educational level of parents varied significantly, with the larger proportion being graduates of high schools (Lyceum) and Vocational Training Schools, i.e., 12 years of education (49.9% of fathers and 50.3% of mothers).

### 3.2. Anthropometric Characteristics—Prevalence of Overweight, Obesity and Abdominal Obesity

The mean BMI and weight were 20.36 ± 3.58 kg/m^2^ and 45.65 ± 10.58 kg, respectively. No statistically significant difference was observed between the two genders, both for BMI [t(1927) = −0.24, *p* = 0.807],), and for weight [t(1927) = −1.49, *p* = 0.136].

However, statistically significant differences were found in the following anthropometric characteristics between genders. Girls were taller (*p* = 0.003) and had a smaller waist circumference (*p* < 0.001), while boys had higher values in zBMI (*p* < 0.001) and the Waist-to-height Ratio (WHR) (*p* < 0.001), as seen in Table 1.

In 50.5% of the sample, bodily weight was normal, while 31.5% of participants had overweight, 17% had obesity, and only 1% were underweight. Statistically significant differences were observed between the two genders, with the prevalence of overweight and obesity being higher in boys compared to girls (overweight 32.6% vs. 30.4% and obese 20.2% vs. 14.2%; χ^2^ (3.1929) = 17.14, *p* = 0.001). In addition, prevalence of abdominal obesity (assessed by WtH Ratio) was 20.3%, being significantly higher in boys (22.8% vs. 18.0% in girls) [χ^2^ (1.1929) = 6.90, *p* = 0.009], (Table 1).

### 3.3. Physical Activity of Children

The preference for activities involving movement was evaluated using a Likert scale. This was found to be very high in general (Median = 9), with the majority (67.4%) having answered 9 or 10 on a scale of 1–10. A statistically significant difference between the two genders was observed, with males having a higher degree of preference (Median = 10 vs. median = 9) [U (907.1022) = 440256.50, z = −2.03, *p* = 0.042].

In line with this, girls reported a higher percentage (compared to boys) of participation in an organized physical activity of less than an hour daily (including those with no participation at all). On the other hand, a larger number of boys participated in organized physical activities for more than two hours/day. This difference between genders was observed on schooldays as well as on weekends (*p* < 0.001 in both circumstances), but not during holidays. The same pattern was observed when participation in free play was asked, with boys devoting a larger proportion of their time on schooldays (*p* = 0.009), at weekends (*p* = 0.001), and during the holiday season (*p* = 0.010).

### 3.4. Physical Activity of Parents

The questionnaire also explored the physical activity of the parents, with 27.2% and 23.8% of the mothers and fathers, respectively, stating that they exercise 3–6 times a week or daily, while a higher proportion, namely similar percentages of both parents (37.7% of mothers and 39.1% of fathers), exercised 1–2 times a week or only on weekends. Furthermore, a significant proportion, namely 35.1% and 37.1%, answered that they did not exercise at all or almost not at all. Mothers were more likely to exercise in gyms (25.3% vs. 12.2% in fathers), while fathers preferred outdoor or park sports (28.5% vs. 21.4% of mothers) or court sports or swimming pools (17.8% vs. 7.3%). Still, the majority of parents (46% of mothers and 41.5% of fathers) preferred their home as a place for exercising physically.

### 3.5. Daily Physical Activity and BMI of Children

Among those that did not participate in organized physical activity (0 h/day), higher rates of obesity (24.9% for boys and 19.0% for girls) were observed, in comparison to those who participated for at least one hour per day. However, the observed differences were not statistically significant for both genders, as seen in Table 2

Also, the hours spent per day in free play were not statistically significantly correlated with the BMI-based subgroups for both boys (*p* = 0.829) and girls (*p* = 0.164). Although, a trend for higher rates of obesity was observed in girls reporting more than two hours of free play each school day. This trend was not observed in boys.

Regarding abdominal obesity, WtH ratios were higher among children who did not participate in any organized activity (30.6% and 22.0% for both genders). These differences were found to be statistically significant only for boys (*p* = 0.015) but not for girls (*p* = 0.123). In line with a previously mentioned finding, in girls who spent at least two hours/day in free play (and not in organized athletic activities) a higher prevalence of abdominal obesity was found (*p* = 0.043). However, free play was not associated with abdominal obesity in boys (*p* = 0.700).

Following that, two multivariate logistic regression models were applied to assess the effect of the examined factors on the occurrence of overweight/obesity (i.e., BMIaz ≥ 1) and of abdominal obesity (WtHR ≥ 0.5). More specifically, the following variables were entered: gender, residence, educational level of father/mother, frequency of physical exercise of father/mother, participation in organized physical activities, participation in free play. Both models resulted in statistical significance (χ^2^(9) = 27.49, *p* = 0.001 for overweight/obesity and χ2(9) = 27.03, *p* = 0.001 for abdominal obesity).

As seen in Table 3, the probability for overweight/obesity is higher in boys (OR = 1.39, 95% CI = 1.16–1.66) compared to girls. Children whose fathers had a higher educational level were less probable to be overweight/obese (OR = 0.75, 95% CI = 0.60–0.93). On the contrary, a higher educational level of the mother was associated with a higher probability of children being in the overweight/obese group (OR = 1.29, 95% CI = 1.05–1.59).

Abdominal obesity was more probable in boys (OR = 1.37, 95% CI = 1.10–1.72). Additionally, children participating at least one hour/day in an organized physical activity had a significantly lower probability for abdominal obesity (compared to those with no physical activity (OR = 0.66, 95% CI = 0.51–0.85). Moreover, children whose fathers exercised at least 1–2 times/week had less probability for abdominal obesity (OR = 0.76, 95% CI = 0.591–0.98).

### 3.6. Organized Physical Activity and Free Play—Place of Residence

Based on the place of residence, no statistically significant difference was observed between the hours of participation in organized physical activity during schooldays, for the total sample [χ^2^ (4.1929) = 6.27, *p* = 0.180], and for the two genders separately [(χ^2^ (4.907) = 0.54, *p* = 0.969; for boys) and (χ^2^ (4.1022) = 8.48, *p* = 0.075; for girls)]

On the contrary, both boys and girls living in urban settings reported a higher degree of no participation in free play at all vs. those living in rural areas. This difference was statistically significant [for boys (χ^2^ (4.907) = 13,64, *p* = 0.009 and for girls χ^2^ (4.1022) = 18.60, *p* = 0.001]. More specifically, the rates of non-participation (0 h) in free play were for boys (21.8% in urban vs. 9.8% in rural settings) and girls (26.4% in urban vs. 11.9% in rural settings). During the holiday period, however, no statistically significant difference was observed either in free play or in organized athletic activities

### 3.7. Organized Physical Activity and Free Play—Educational Level of Parents

The hours of participation of children in organized physical activity, during the school days, were significantly different according to the educational level of their parents [(χ^2^ (6.1929) = 74.33, *p* < 0.001) and (χ2 (6.1929) = 81.29, *p* < 0.001) of father and mother respectively]. In particular, very few children of parents with high educational backgrounds did not participate in organized activities (0 h/day). This was reported by 14.5% of children with a father holding a Postgraduate/doctoral degree and 10.2% with a mother with the same educational level.

On the contrary, children of highly educated parents did not usually participate in free play. This was observed both for highly educated fathers [χ^2^ (6.1929) = 72.94, *p* < 0.001] and mothers [χ^2^ (6.1929) = 50.21, *p* < 0.001].

Likewise, during weekends, hours spent in organized physical activity were significantly different both for different educational levels of the father [χ^2^ (6.1929) = 35.47, *p* < 0.001], as well as that of the mother [χ^2^ (6.1929) = 29.06, *p* < 0.001]. During the holidays, no statistically significant difference was observed.

## 4. Discussion

This study aimed to capture the physical activity and bodily characteristics, such as BMI and abdominal obesity, in a large sample of pre-adolescents, in the context of their socio-demographic characteristics in the region of Thrace, NE Greece. Thrace is a region characterized by cultural diversity, and previous studies have tried to explore health indices among children’s populations. According to Glania et al., among high school children, younger boys, non-natives, and those whose parents received higher education were at greater risk of being injured [22]. Also, the prevalence of caries was studied in the region and was found to be high in general due to environmental factors, independent of various socio-demographic factors [23].

In our study population, the prevalence of overweight and obesity was non-neglectable (48.5%) and was observed more frequently in male pre-adolescents. As expected, obesity rates were higher among those not participating in physical activities. In general, participation in any type of physical activity during school days was >75%, with significant differences being observed according to the place of residence and the educational level of the parents.

The degree of preference in activities involving movement was found to be very high, with males having a higher degree of preference compared to females. A comprehensive school physical activity program provides opportunities for moderate to vigorous physical activity (MVPA) before, during, and after school. Physical activity is practiced through the formal teaching of sports, and informally through recesses and/or active transportation to and from school [24]. The school environment plays a protective role in the occurrence of adverse health effects. The key element that distinguishes weekends from summer days is the prolonged and concentrated period when children are exposed to a less structured environment [25]. Primary schools seem to be an ideal environment for implementing holistic obesity prevention strategies, with physical education being the best tool [26]. The World Health Organization (WHO) guidelines for physical exercise are at least 60 min MVPA per day, with Germany being the only country to advise at least 90 min per day [27], and participating at least three times a week in activities that enhance muscles and bones to benefit the health of children and adolescents [28,29]. As applied in Greece, children in Bosnia and Herzegovina, due to the school curriculum, only have 45 min of physical activity twice a week, which according to researchers is certainly not enough to bring about the benefits related to health and fight against obesity [30].

In our study, on school days, 76.1% of the total sample participated in at least 1 h/day in organized physical activity, and 78% in free play. However, few young people meet the national guidelines. Children and young people (5–17 years old) do not achieve the 60 min MVPA per day [28,31]. In our study, declining participation of children in organized physical activity and an increase in free play on weekends was observed, which was more overt during the holidays. Differences in physical activity over the weekends suggest that a greater focus on how the weekend activity contributes to the average of 60 min MVPA per day during the week may be justified [3].

Regarding differences between genders, in a previous study from China, boys reported higher levels of MVPA. They were more likely to respond to the 60 min MVPA recommendations per day than their female counterparts [32]. In the present study, in organized physical activity, the boys participated for at least 2 h/day while the girls were more active in the non-participation groups. In the study of Lu et al., also from China, boys were consistently more active than girls [33]. Also, in the study of Jalali-Farahani et al. from Iran, both during school and during the holiday season, the average weekly time in sports activities was significantly higher in boys [34].

The interventions could be oriented from a social perspective that includes the families and networks of close friends [35]. Increased participation in organized physical activity at school and in the community is associated with greater overall physical activity [36]. The choice of extracurricular sports can be another factor that influences the increase of exercise and the provision of an active lifestyle in children [37]. High levels of sedentary behavior have been associated with negative health indicators, including obesity [38], while higher levels of overall physical activity and MVPA were associated with lower BMI [39]. In line with this, our major finding was the highest percentage of obesity for both genders, but especially for boys, with no physical activity during the day. No statistical significance was observed, however, between hours of exercise and BMI. This was probably due to other confounding factors, such as nutritional habits.

Changes in economic status and socio-demographic profiles associated with urbanization lifestyle behaviors transition to unhealthy eating patterns and less physical activity [40]. The place of residence determines the conditions for free play or organized activity [33]. Parents should encourage children to attend sports clubs after school and to play at home and in the neighborhood [36]. In our study, the percentage of girls who lived in rural areas that did not participate in organized physical activity was higher, as significantly higher were the rates of non-participation in free play for both boys and girls living in urban areas.

Socio-economic status is positively associated with health standards. Higher levels of parenting education are associated with high participation in physical activity, while lower levels are associated with high sedentary behavior [41]. The study by Romero-Blanco et al. emphasizes the positive correlation of a supportive family environment with the practice of extracurricular sports [42]. In the present study, among children whose parents were graduates of primary school/Gymnasium, the percentage of participation only in free play (and not in organized activities) was significantly higher, while free play was less frequent among children with parents of higher educational level.

Childhood obesity is considered one of the most serious global public health challenges of the 21st century [43]. Children have distinct behavioral profiles influenced by gender, weight status, socio-economic status, parents’ occupational condition [13,17]. Adolescents with obesity are at increased risk for many adverse health conditions, including depression, physical dissatisfaction, overeating, and engaging in disturbed weight control behaviors [44]. The prevalence of overweight and obese young people in both developed and underdeveloped countries has increased by approximately 47% in the last 10 years [45]. According to WHO, it is expected that if the current trend continues, in 2025 there will be 20% or more obese children and adolescents in more than 30 countries around the world [46,47]. Support for preventive health care and the timely development of appropriate dietary and lifestyle behaviors is essential [48]. Knowledge of health, attitudes, and behavior is acquired during childhood and adolescence. [49]. Behavior-modifying multi-component interventions may be beneficial in achieving small, short-term reductions in BMI, z- BMI, and weight in children aged 6 to 11 years [50].

In our study, 48.5% of participants were either overweight or obese, while only 1.0% belonged to the underweight category. In the study of Jalali-Farahani et al., 38.5% of children in Iran were overweight or obese, 58.7% had normal weight, and 2.8% were thin or very thin [34]. In the study by Horodyska et al., 24.3% of children in Poland were overweight/obese, and 10.1% were underweight [51].

The prevalence of obesity in 2013–2016 in US young people aged 2–19 years was 17.8%, and the prevalence of severe obesity was 5.8% [52]. According to our study, the prevalence of overweight and obesity was higher in boys than girls. Likewise, in the study of Wang et al., boys in China are twice as likely to be overweight-obese as girls and are more prevalent in urban areas than in rural areas [53]. Also, in the study by Ferranti et al., boys in Italy had a higher rate of overweight and obesity than girls, and in adolescence were significantly more likely to become overweight and obese [54]. In the study of Min et al., from China, boys, young children (<12 years old), and rural children had higher BMI than girls, older children, and urban children [55]. According to another study from Bosnia, the prevalence of abdominal obesity was 19% and 13% among boys and girls, respectively. Compared to boys, girls were shorter and had a lower waist circumference [30].

In our study, girls were taller and had a lower waist circumference, while boys had higher values in both BMIaz and WHtR. In addition, the prevalence of abdominal obesity was significantly higher in boys (22.8%) compared to girls (18.0%). Logistic regression analysis revealed that the male gender was associated with a higher probability of overweight/obesity and abdominal obesity, even though boys declared a higher preference for activities involving physical exercise. A finding worth noting, however, is the higher prevalence of abdominal obesity in those girls who spent more than two hours/day outside the home in free play (but not in organized activities). A possible explanation is the lack of parental supervision, which may result in unhealthy nutritional habits.

Promoting physical activity among young people is considered a priority to facilitate the transfer of an active lifestyle to adulthood [14]. The frequency of parental exercise is a role model for children. Modeling roles with the active participation of parents, providing logistical support and creating a healthy home environment, affects the weight status of children, especially in the first years of life [56,57]. There is an enhancing feedback cycle between parents and children that mutually influences each other’s behavior [14,58].

In the study by Ogden et al., the prevalence of obesity among young people living in households with a head of household with a high school diploma or less (22.3%) or in households with a college education (18.1%) was significantly higher compared with young people living in households headed by college graduates (11.6%), [52]. In the study by Ferranti et al., it was found that adolescents who had parents with higher education or a specialized occupation were more likely to be underweight or have normal weight [54]. In our study sample, the educational background of the father was associated with a lesser probability for overweight/obesity, a finding not confirmed regarding the mother’s educational background.

At the individual and interpersonal level, parents and the home environment affect the development of the child’s attitudes, beliefs, knowledge, and behavior [58]. Parents should help children develop proper body image relative to maintaining a healthy body weight [2,55]. Physical activity and its promotion can be valuable tools for controlling children’s body weight and improving their long-term health [59].

## 5. Conclusions

In conclusion, lack of physical exercise is associated with a higher prevalence of obesity for both boys and girls in pre-adolescence. Socio-demographic factors promoting physical activity, either in organized or free mode, can play a significant role in establishing physical activity as a way of life. Weekends and holidays are very good times to make targeted interventions aimed at increasing participation in organized activities, encouraging benefits in physical as well as in mental health.

## Figures and Tables

**Table 1:** Anthropometric characteristics of the sample by gender.

		Gender		
Feature	Total(N = 1929)	Boys(n = 907)	Girls(n = 1022)	*p*-Value
	Mean ± SD	Mean ± SD	Mean ±S D	
Weight (kg)	45.65 ± 10.58	45.27 ± 10.44	45.99 ± 10.70	*p* = 0.136
Height (m)	1.49 ± 0.08	1.49 ± 0.08	1.50 ± 0.08	*p* = 0.003
Waist circumference (cm)	67.59 ± 9.38	68.57 ±9.50	66.73 ± 9.19	*p* < 0.001
BMI (kg/m^2^)	20.36 ± 3.58	20.34 ±3.47	20.38 ± 3.66	*p* = 0.807
zΒΜΙ	0.88 ± 1.13	1.00 ± 1.13	0.77 ± 1.12	*p* < 0.001
WtHR	0.45 ± 0.06	0.46 ± 0.05	0.44 ± 0.05	*p* < 0.001
BMI classification	n (%)	n (%)	n (%)	
Underweight	19 (1.0)	8 (0.9)	11 (1.1)	*p* = 0.001
Normal weight	975 (50.5)	420 (46.3)	555 (54.3)	
Overweight	607 (31.5)	296 (32.6)	311 (30.4)	
Obese	328 (17.0)	183 (20.2)	145 (14.2)	
Abdominal obesity	n (%)	n (%)	n (%)	
Yes ( WtHR ≥ 0.5)	391 (20.3)	207 (22.8)	184 (18.0)	*p* = 0.009
No ( WtHR < 0.5)	1538 (79.7)	700 (77.2)	838 (82.0)	

**Table 2:** Comparison of hours spent in organized physical activity and free play, based on BMI- and abdominal obesity groups analyzed by gender.

	Boys (n = 907)				Girls (n = 1022)			
	Organized Physical Activity				Organized Physical Activity			
	0 h (n = 193)	1 h (n = 354)	≥2 h (n = 360)		0 h (n = 268)	1 h (n = 474)	≥2 h (n = 280)	
Groups based on ΒΜΙ	*n (%)*	*n (%)*	*n (%)*	*p*-value	*n (%)*	*n (%)*	*n (%)*	*p*-value
Normal weight	91 (47.2%)	174 (49.2%)	163 (45.3%)	*p* = 0.250	136 (50.7%)	263 (55.5%)	167 (59.6%)	*p* = 0.067
Overweight	54 (28.0%)	116 (32.8%)	126 (35.0%)		81 (30.2%)	151 (31.9%)	79 (28.2%)	
Obese	48 (24.9%)	64 (18.1%)	71 (19.7%)		51 (19.0%)	60 (12.7%)	34 (12.1%)	
Abdominal obesity	*n (%)*	*n (%)*	*n (%)*	*χ^2^(df,N)* *p*-value	*n (%)*	*n (%)*	*n (%)*	*χ^2^(df,N)* *p*-value
Yes (WHR ≥ 0.5)	59 (30.6%) ^a^	75 (21.2%) ^b^	73 (20.3%)	*p* = 0.015	59 (22.0%)	81 (17.1%)	44 (15.7%)	*p* = 0.123
No (WHR < 0.5)	134 (69.4%) ^a^	279 (78.8%) ^b^	287 (79.7%) ^b^		209 (78.0%)	393 (82.9%)	236 (84.3%)	
	**Free play**				**Free play**			
	**0 h** **(n = 179)**	**1 h** **(n = 301)**	**≥ 2 h** **(n = 427)**		**0 h** **(n = 245)**	**1 h** **(n = 372)**	**≥ 2 h** **(n = 405)**	
Groups based on ΒΜΙ	*n (%)*	*n (%)*	*n (%)*	*p*-value	*n (%)*	*n (%)*	*n (%)*	*p*-value
Normal weight	88 (49.2%)	143 (47.5%)	197 (46.1%)	*p* = 0.829	141 (57.6%)	217 (58.3%)	208 (51.4%)	*p* = 0.164
Overweight	52 (29.1%)	99 (32.9%)	145 (34.0%)		77 (31.4%)	102 (27.4%)	132 (32.6%)	
Obese	39 (21.8%)	59 (19.6%)	85 (19.9%)		27 (11.0%)	53 (14.2%)	65 (16.0%)	
Abdominal obesity	*n (%)*	*n (%)*	*n (%)*	*p*-value	*n (%)*	*n (%)*	*n (%)*	*p*-value
Yes (WtHR ≥ 0.5)	45 (25.1%)	68 (22.6%)	94 (22.0%)	*p* = 0.700	38 (15.5%)	58 (15.6%)	88 (21.7%)	*p* = 0.043
No (WtHR < 0.5)	134 (74.9%)	233 (77.4%)	333 (78.0%)		207 (84.5%)	314 (84.4%)	317 (78.3%)	

**Table 3:** Logistic regression analysis models for overweight/obesity (BMIaz ≥1) and abdominal obesity (WtHR ≥ 0.5).

		Overweight/Obesity			Abdominal Obesity		
Indipendent variables	N	OR	95% CI	*p*-value	OR	95% CI	*p*-value
Gender							
Girls	1022	(Ref.)	(Ref.)	(Ref.)	(Ref.)	(Ref.)	(Ref.)
Boys	907	1.39	(1.16–1.66)	*p* < 0.001	1.37	(1.10–1.72)	*p* = 0.006
Residence							
Urban settings	1463	(Ref.)	(Ref.)	(Ref.)	(Ref.)	(Ref.)	(Ref.)
Semi-urban settings	225	0.86	(0.64–1.14)	*p* = 0.287	0.87	(0.61–1.25)	*p* = 0.461
Rural settings	241	0.99	(0.75–1.13)	*p* = 0.920	0.86	(0.61–1.22)	*p* = 0.406
Education level of the father							
High school graduates/Vocational training	1238	(Ref.)	(Ref.)	(Ref.)	(Ref.)	(Ref.)	(Ref.)
University- Post-graduate studies	691	0.75	(0.60–0.93)	*p* = 0.009	0.91	(0.70–1.19)	*p* = 0.511
Education level of the mother							
High school graduates/Vocational training	1133	(Ref.)	(Ref.)	(Ref.)	(Ref.)	(Ref.)	(Ref.)
University- Post-graduate studies	796	1.29	(1.05–1.59)	*p* = 0.015	1.14	(0.88–1.47)	*p* = 0.320
Physical exercise of the father (frequency)							
Not at all/Almost not at all	715	(Ref.)	(Ref.)	(Ref.)	(Ref.)	(Ref.)	(Ref.)
1–2 times/week or more	1214	0.95	(0.77–1.16)	*p* = 0.606	0.76	(0.59–0.98)	*p* = 0.032
Physical exercise of the mother (frequency)							
Not at all/Almost not at all	678	(Ref.)	(Ref.)	(Ref.)	(Ref.)	(Ref.)	(Ref.)
1–2 times/week or more	1251	0.89	(0.73–1.10)	*p* = 0.282	0.93	(0.72–1.20)	*p* = 0.586
Participation in organized physical activities							
O hours/day	461	(Ref.)	(Ref.)	(Ref.)	(Ref.)	(Ref.)	(Ref.)
1 h/day or more	1468	0.89	(0.72–1.10)	*p* = 0.283	0.66	(0.51–0.85)	*p* = 0.001
Participation in free play							
0 h/day	424	(Ref.)	(Ref.)	(Ref.)	(Ref.)	(Ref.)	(Ref.)
1 h/day or more	1505	1.10	(0.88–1.37)	*p* = 0.427	1.02	(0.77–1.35)	*p* = 0.896

OR = Odds Ratio, CI = Confidence Interval, Ref. = Reference Category.

## Data Availability

The data presented in this study are available on request from the corresponding author.
